# Living methods for living guidelines: Changes to evidence synthesis methods in the Australian National Clinical Evidence Taskforce COVID‐19 living guidelines

**DOI:** 10.1002/gin2.12017

**Published:** 2024-05-21

**Authors:** Heath White, Tari Turner, Claire Beecher, Alex Poole, Steve McDonald, Jessie Hewitt, Samantha Chakraborty, David Fraile‐Navarro, Saskia Cheyne, Joshua Vogel, Britta Tendal Jeppesen, Steve McGloughlin, National Clinical Evidence Taskforce

**Affiliations:** ^1^ School of Public Health and Preventive Medicine Monash University Melbourne Victoria Australia; ^2^ Burnet Institute Melbourne Victoria Australia; ^3^ Future Evidence Foundation Melbourne Victoria Australia; ^4^ Alfred Health Melbourne Victoria Australia

**Keywords:** COVID‐19, guideline methods, living evidence, living guidelines

## Abstract

Living evidence methods, such as those used to produce living guidelines, can evolve over time as the context or evidence changes. In Australia, the National Clinical Evidence Taskforce has been developing living guidelines for the management and care of people with coronavirus disease 2019 (COVID‐19) since March 2020, undertaking daily searches, and producing over 130 updates of more than 200 recommendations. Over the 3 years of the guidelines, the methods have also been ‘living’. In this paper, we describe why, how and with what impact changes to our methods have been made. When changes were required to the methods, the Taskforce Evidence Team developed a ‘Methods Brief’ outlining the proposed changes, rationale and any risks. This was presented to the Guidelines Leadership Group for approval and to the Steering Committee for noting. Changes were then reflected in the online, publicly available description of our methods. Methods to develop the living guidelines evolved through five phases, reflecting changes in the availability of evidence, the degree and nature of clinical uncertainty and resource availability. Largely these changes were to the criteria we used to select evidence for inclusion, and our expected level of responsiveness to new evidence. In the initial phases, inclusion criteria were very broad, and as the evidence base stabilised our focus narrowed to areas of high clinical importance and evidence certainty. The rapidly evolving nature of the pandemic, understanding of the illness, clinical questions and evidence base during development of the living COVID‐19 guidelines, necessitated multiple changes to the methods used to produce the guidelines. In this context, the ongoing revision of the methods for living guideline production was a necessity and a strength of the living approach. Questions remain about how best to ensure rigour is maintained while methods evolve.

## Key points

**What is already known on this topic:**

Living guidelines are a valuable, feasible way to keep clinicians up‐to‐date with emerging evidence.

**What this study adds:**

Changes to the methods of living guidelines to reflect changes in the context or nature of the evidence can be made transparently and effectively.

**How this study might affect research, practice or policy:**

Our experience provides support for living approaches and raises important questions about opportunities for bias.

## 1 INTRODUCTION

Living evidence methods, including those used to produce living systematic reviews and living guidelines, are designed to ensure that these evidence syntheses are kept up‐to‐date as new research evidence emerges. One potential benefit of the iterative nature of living syntheses is that their methods can evolve to reflect changes in the clinical or policy context, or in the rate or nature of the evidence being produced.

While there has been rapid global uptake of living approaches,^1^, ^2^ particularly since the onset of the coronavirus disease 2019 (COVID‐19) pandemic, the field of living evidence synthesis is reasonably nascent, and there are limited experiences of how changes to evidence synthesis methods play out over time in living reviews or guidelines, and the implications, or acceptability of these changes.

In Australia, the National Clinical Evidence Taskforce has been developing living guidelines for the management and care of people with COVID‐19 since March 2020.^3^ The Taskforce guidelines are an example of extremely rapid living guidelines, with searches for new evidence conducted daily, and guideline updates made as often as weekly.^4^ Since their establishment the guidelines have been updated more than 130 times and grown to include over 200 recommendations.

During the 3 years of the guidelines, the degree of uncertainty in the clinical context, the nature of the clinical questions, the availability of evidence and the virus itself have altered rapidly and substantially. The pandemic provided both the need and opportunity to repeatedly revise and refine the living synthesis methods employed and to examine the feasibility and acceptability of living guideline development methods in a real‐world context.

## 2 CHANGES TO GUIDELINE DEVELOPMENT METHODS

The GRADE‐based methods used to develop and maintain the Taskforce COVID‐19 guidelines have been described previously.^3^ Potentially relevant studies identified from daily surveillance of PubMed, select preprint servers and key journals are assessed by the evidence review team that creates evidence profiles, evidence‐to‐decision tables and evidence summaries in MAGICapp, and draft recommendations for review by multidisciplinary panels at online meetings. Once developed by a clinical panel, new and updated recommendations are approved by the Guideline Leadership Group and Steering Committee before publication. While core methods have been stable over time (use of evidence‐based GRADE methods, which meet National Health and Medical Research Council standards for approval), the application of these methods has evolved during different phases of the guidelines.

When changes were required to the methods, these were discussed and agreed upon by the Taskforce Evidence Team, which then developed a ‘Methods Brief’ outlining the proposed changes, rationale and any potential risks. The Methods Brief was presented to the Guidelines Leadership Group for discussion and approval and, once approved, to the Steering Committee for noting. Changes were then reflected in the online, publicly available description of our methods,^5^ and the technical documentation. Table 1 represents an overview of these changes.

**Table 1:** Overview of methodological changes.

Phase	Impetus for change	Process	Impact
Phase 1	High novelty of condition with limited direct evidence	Analysis of existing guidelines and indirect evidence (e.g., SARS/MERS)	Development of initial consensus recommendations
Phase 2	Early comparative evidence (primarily observational) published	All relevant evidence identified used to develop (direct) evidence base	Primarily ‘only in research’ recommendations developed (‘more evidence needed’)
Phase 3	Significant body of evidence published in peer‐reviewed journals and preprint servers	Analysis of all RCT evidence for drug treatments, and other evidence where RCT data is not possible/feasible (e.g., timing of surgery following infection)	Explosion in the number of recommendations (primarily ‘only in research’ due to low/very low certainty evidence); first strong/conditional recommendations developed
	Abundance of small RCTs published focused on various experimental treatments	Thresholds for analysis determined; studies added to ‘surveillance’ if considered insufficient for the development of impactful recommendation	Reduction in the number of new ‘only in research’ recommendations; modifications to impactful recommendations prioritised
Phase 4	Significant body of evidence was published; however, generally focused on nonimpactful topic areas, or impactful areas with an already strong evidence base	Topics classified as high, medium and low priority; analysis of high impact evidence for high (and some medium) priority topics only (remainder added to ‘surveillance’ list)	Few new ‘only in research’ recommendations; focus on updating impactful/clinically important recommendations only
Phase 5	Reduction in funding, limited publication of new high‐impact evidence	Analysis of high‐impact evidence only (remainder added to ‘surveillance’ list)	No new recommendations unless significant evidence is identified; modifications to existing impactful/clinically important recommendations only

Abbreviations: MERS, Middle East respiratory syndrome; RCT, randomised controlled trials; SARS, severe acute respiratory syndrome.

### 2.1 Phase 1: ‘I want it all and I want it now’—Overwhelming urgency and uncertainty, no evidence (March–April 2020)

During the first phase of the pandemic, and of the guideline development, there was an overwhelming urgency to rapidly develop guidance for very concerned clinicians who were expecting to treat an imminent influx of people with COVID‐19. In this period there was significant clinical uncertainty—no one had treated COVID‐19 previously, and no direct research evidence was available to underpin evidence‐based recommendations.

In this initial phase, the Taskforce guidelines consisted entirely of consensus recommendations developed largely through adaptation from other national and international guidelines for similar conditions or symptoms, for example, severe acute respiratory syndrome or Middle East respiratory syndrome. In this phase, recommendations largely affirmed the need to continue routine evidence‐based practice while waiting for high‐quality direct evidence to become available for COVID‐19.

This approach enabled the Taskforce to rapidly provide nationally consistent guidance, providing reassurance and clarity to Australian clinicians.

### 2.2 Phase 2: ‘Under pressure’—All comparative evidence is good evidence (April–September 2020)

As COVID‐specific evidence became available moving into the second half of 2020, the Taskforce initially included all comparative evidence addressing clinical questions within the scope of the guidelines (management and care of people with COVID‐19).

Early on we decided to focus on areas of management that were likely to be different from usual practice (i.e., we would not make recommendations that were for usual care of patients with respiratory illnesses, but only where care should be different for patients with COVID‐19).

In this phase, we included any comparative evidence that examined interventions to treat COVID‐19, including small observational studies, because the data available to inform clinical decision‐making was very limited and it was unclear which interventions were effective. As COVID‐19 research output gathered pace throughout 2020, more robust evidence became available, prompting a new approach.

### 2.3 Phase 3: ‘You don't fool me’—Focusing on the best available evidence (September 2020–July 2021)

The increasing rate of publication of RCTs, identified through daily searches of peer‐reviewed databases and the preprint servers medRxiv and Research Square, was a turning point in Taskforce guideline methods. In this phase, we restricted our focus to the inclusion of RCTs of interventions, with minimal exceptions where it was unlikely, infeasible or unethical to conduct RCTs (such as therapies for existing indications or timing of surgery).

Initially, trials of new treatments, irrespective of size or quality, were assessed and a recommendation (typically ‘only in research’) was included in the guideline. During this phase, living evidence platforms, such as the COVID‐NMA initiative,^6^ were a useful supplementary resource to crosscheck study data and risk of bias assessments. As the flow of trials increased, we further restricted our synthesis efforts to those trials that either individually or collectively provided sufficient evidence to inform an evidence‐based recommendation to use or not use a particular treatment.

### 2.4 Phase 4: ‘Don't stop me now’—Prioritising areas of uncertainty (July 2021–December 2022)

As the evidence base stabilised, we assigned priority levels to our growing list of recommendations: low‐priority recommendations were unlikely to be updated; moderate priority would be updated on the advice of our Guidelines Leadership Group, and high priority would continue to be updated as new evidence emerged. These categories were assigned based on the clinical importance of the recommendation (based on Taskforce consensus), and the likelihood that new evidence would change the recommendation. For example, prompt inclusion of new evidence into the existing evidence base underpinning the conditional recommendation supporting the use of remdesivir was considered a high priority, whereas the inclusion of new evidence into less impactful recommendations with limited evidence, for example, triazivirin and bromhexine hydrochloride, was considered low priority.

In this period, we also began to investigate the use of in vitro data to revise recommendations, as new variants of the virus emerged, and studies suggested that the monoclonal antibody drugs earlier found to be effective in RCTs conducted with earlier strains of SARS coronavirus‐2 were no longer neutralising new variants in the laboratory. But that is a whole other paper.

### 2.5 Phase 5: ‘Who wants to live forever’—Focusing on clinically relevant changes (January 2023–)

At the end of 2022, government funding for the Taskforce was discontinued. With very limited resources to maintain the guidelines, our focus is now on incorporating new evidence only where it is likely to change the direction or certainty of a recommendation.

New recommendations have also been restricted to areas of the potential impact on clinical practice in Australia (e.g., we no longer make recommendations for drugs that are not available in Australia, unless these are very high‐profile drugs or studies).

## 3 DISCUSSION

The rapidly evolving nature of the pandemic, understanding of the illness, clinical questions and evidence base during development of the living COVID‐19 guidelines, necessitated multiple changes to the methods used to produce the guidelines. In this paper, we have focused on changes to the evidence synthesis methods, but changes were also made to our clinical and consumer panel structure and composition; communication and engagement strategies; approach to issues of equity and diversity and so on.

Changes to methods were also made necessary by our varying funding situation, compelling prioritisation of limited evidence synthesis resources to where they were perceived to have the most benefit. We think this is unlikely to have impacted the reliability or clinical utility of the guidelines and our intention when making any change was to focus on maintaining rigour and addressing the areas of greatest clinical need.

Changes in the context and evidence base that prompted us to revise our methods were to a great degree unpredictable. While we could have foreseen the increasing availability of evidence over time, it would have been very difficult to predict the timing and extent of these changes, or how they would align with our priority questions and clinical needs. The ability to responsively revise our methods was a major strength of our guideline development process, allowing us to adapt as changes arose. It will be interesting to explore the implications of this when planning for similar future projects.

Transparency about the methods used and a clear process for changing these methods were vital for ensuring the reliability and trustworthiness of the guidelines. Clear documentation of the changes made and the rationale is crucial—as became clear when there was external pressure for observational evidence to be used for some recommendations and not others—a pressure we could resist given the transparency and public availability of our approach. This documentation is also important for mechanisms for approval and endorsement of living guidelines—ensuring that approvers can assess the reliability and appropriateness of the methods employed.

Our experience of the COVID‐19 guidelines was that an iterative, responsive approach to revising guideline methods was acceptable to our clinical leadership and our guideline users—healthcare professionals in Australia.^7^ It will be interesting to see whether there is similar acceptance in other clinical contexts.

The COVID‐19 experience was exceptional on many fronts and it remains an open question as to whether this same process of multiple revisions to guideline development methods, and perhaps even similar reasons for changes, will play out in other living guidelines, albeit at a slower pace.

Iterative approaches to methods development are a relatively unexamined element of living evidence synthesis methods. While accepted practice, and a purported strength of this approach, there is a need to explore whether these changes introduce potential biases, and, if so, how these can be minimised and mitigated.

The ongoing, iterative nature of the development of living guidelines lends itself to the incorporation of embedded process evaluation. Such evaluation allows opportunities to proactively identify needs and opportunities for changes to the guideline development methods (as well as other aspects of the process including scope, dissemination, etc.^8^).

## 4 CONCLUSION

When developing a rapid, living guideline in an emergent context such as the COVID‐19 pandemic, the iteration of the synthesis methods for living guideline production was a necessity and a strength of the living approach. There are many questions about how to do this well; ensuring that rigour is maintained, that living guidelines remain viable long‐term and that all involved parties understand its iterative nature.

## AUTHOR CONTRIBUTIONS

Tari Turner and Heath White prepared the first draft of this manuscript, which all authors reviewed and revised. All authors developed the concepts presented in this manuscript. All authors read and approved the final manuscript. The authors were all involved in the development and application of the methods described herein.

## CONFLICT OF INTEREST STATEMENT

The authors declare no conflict of interest.

## ETHICS STATEMENT

Ethics statement was not required for this article. All authors approve this manuscript for publication.

## Data Availability

Data sharing is not applicable to this article as no data sets were generated or analysed during the current study.
